# Epimutations of the IG-DMR and the *MEG3*-DMR at the 14q32.2 imprinted region in two patients with Silver–Russell Syndrome-compatible phenotype

**DOI:** 10.1038/ejhg.2014.234

**Published:** 2014-11-05

**Authors:** Masayo Kagami, Seiji Mizuno, Keiko Matsubara, Kazuhiko Nakabayashi, Shinichiro Sano, Tomoko Fuke, Maki Fukami, Tsutomu Ogata

**Affiliations:** 1 Department of Molecular Endocrinology, National Research Institute for Child Health and Development, Tokyo, Japan; 2 Department of Pediatrics, Central Hospital, Aichi Human Service Center, Aichi, Japan; 3 Department of Maternal-Fetal Biology, National Research Institute for Child Health and Development, Tokyo, Japan; 4 Department of Pediatrics, Hamamatsu University School of Medicine, Hamamatsu, Japan

## Abstract

Maternal uniparental disomy 14 (UPD(14)mat) and related (epi)genetic aberrations affecting the 14q32.2 imprinted region result in a clinically recognizable condition which is recently referred to as Temple Syndrome (TS). Phenotypic features in TS include pre- and post-natal growth failure, prominent forehead, and feeding difficulties that are also found in Silver–Russell Syndrome (SRS). Thus, we examined the relevance of UPD(14)mat and related (epi)genetic aberrations to the development of SRS in 85 Japanese patients who satisfied the SRS diagnostic criteria proposed by Netchine *et al* and had neither epimutation of the *H19*-DMR nor maternal uniparental disomy 7. Pyrosequencing identified hypomethylation of the *DLK1-MEG3* intergenic differentially methylated region (IG-DMR) and the *MEG3*-DMR in two cases. In both cases, microsatellite analysis showed biparental transmission of the homologs of chromosome 14, with no evidence for somatic mosaicism with full or segmental maternal isodisomy involving the imprinted region. FISH and array comparative genomic hybridization revealed neither deletion of the two DMRs nor discernible copy number alteration in the 14q32.2 imprinted region. Methylation patterns were apparently normal in other six disease-associated DMRs. In addition, a thorough literature review revealed a considerable degree of phenotypic overlap between SRS and TS, although body asymmetry was apparently characteristic of SRS. The results indicate the occurrence of epimutation affecting the IG-DMR and the *MEG3*-DMR in the two cases, and imply that UPD(14)mat and related (epi)genetic aberrations constitute a rare but important underlying factor for SRS.

## Introduction

Human chromosome 14q32.2 harbors an imprinted region with several paternally expressed genes such as *DLK1* and *RTL1* and maternally expressed genes such as *MEG3* and *RTL1as*, together with the germline-derived primary *DLK1*-*MEG3* intergenic differentially methylated region (IG-DMR) and the post fertilization-derived secondary *MEG3*-DMR.^1, 2^ Consistent with this, maternal uniparental disomy 14 (UPD(14)mat) results in clinically discernible features such as pre- and post-natal growth failure, characteristic face with prominent forehead and micrognathia, small hands, muscular hypotonia, and precocious puberty.^3^ These UPD(14)mat clinical features are also caused by microdeletions involving paternally derived *RTL1* and/or *DLK1* and by epimutation (hypomethylation) affecting the normally methylated IG-DMR and *MEG3*-DMR of paternal origin.^2, 4, 5, 6, 7^ Recently, such a clinically recognizable condition has been referred to as ‘Temple Syndrome' (TS).^8^

Clinical features of TS partially overlap with those of other imprinting disorders. Indeed, pre- and post-natal growth failure, small hands, and hypotonia during early infancy are also observed in Prader–Willi Syndrome (OMIM 176270),^9^ and UPD(14)mat and epimutations involving the IG-DMR and the *MEG3*-DMR have been identified in several patients diagnosed as having Prader–Willi Syndrome.^5, 7, 10^ Furthermore, pre- and post-natal growth failure, prominent forehead, micrognathia, and muscular hypotonia during early infancy are often found in Silver–Russell Syndrome (SRS) (OMIM 180860).^11^ To our knowledge; however, UPD(14)mat has been identified only in a single patient diagnosed as having SRS with no description of detailed phenotype.^12^

Here, we report on epimutations of the IG-DMR and the *MEG3*-DMR in two patients with SRS-compatible phenotype, and discuss on phenotypic overlap between SRS and TS.

## Patients and methods

### Patients

We studied 85 Japanese SRS patients in whom underlying genetic factors remained unknown from our previous study for 138 SRS patients^13^ who satisfied the mandatory criteria and at least three of the five scoring system criteria proposed by Netchine *et al*^14^ (for details of the criteria, see footnote of Table 1). In the previous study,^13^ we identified *H19*-DMR hypomethylation (epimutation) in 43 patients (31.2%) and UPD(7)mat in nine patients (6.5%), and revealed a microdeletion at chromosome 17q24 in a single patient by analyzing copy number alterations for chromosome 11p15.5, 7p12.2, 12q14, and 17q24 that have been identified in rare SRS patients.^15, 16, 17, 18^

The 85 patients had a less-typical SRS phenotype (for details, see Fuke *et al*^13^). Indeed, of the 85 patients, none showed all of the five Netchine scoring system features, and 19 and 66 patients manifested four and three scoring system features, respectively. By contrast, of the 43 patients with *H19*-DMR epimutations, 10 patients were positive for all the five Netchine scoring system features, and 16 and 17 patients exhibited four and three scoring system features, respectively. This phenotypic difference was primarily due to the difference in the frequencies of relative macrocephaly at birth (35.6% *vs* 100%) and body asymmetry (32.2% *vs* 81.1%) between the two groups; the frequencies of the remaining three scoring system features were similar between the two groups. As our previous study included a large number of such patients with less-typical SRS phenotype, this would explain why the prevalence of *H19*-DMR epimutations was lower in our previous study than in Western European studies reported in the literature.^11, 14, 19^ The phenotypes of the nine UPD(7)mat patients fell between those of the 85 idiopathic SRS patients and those of the 43 epimutation-positive patients, with the frequencies of relative macrocephaly at birth and body asymmetry being 77.8% and 33.3%, respectively. This appeared to be consistent with the prevalence of UPD(7)mat being similar between our previous study and Western European studies.^11, 15, 19, 20, 21^

### Ethical approval and samples

This study was approved by the Institute Review Board Committees of National Center for Child Health and Development and Hamamatsu University School of medicine, and performed using peripheral leukocyte samples after obtaining written informed consent.

### Molecular studies

**Figure 1:**
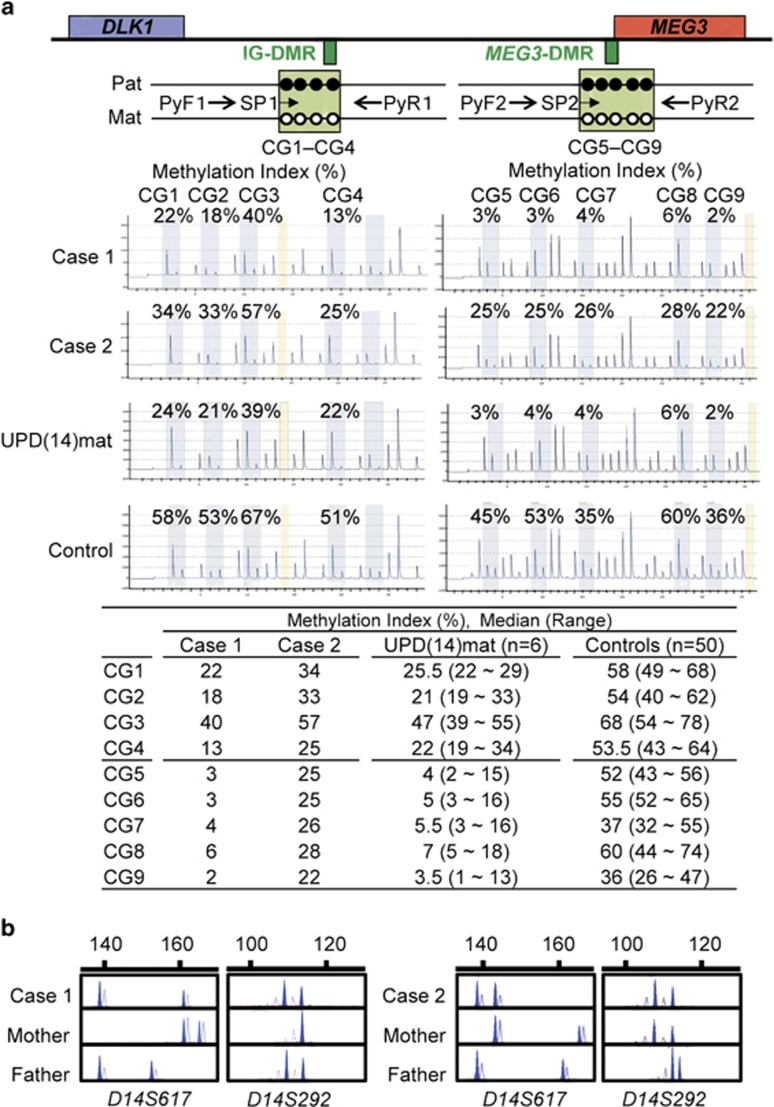
Representative molecular findings. (**a**) Methylation analysis by pyrosequencing analysis. Top panel: schematic representation indicating of four CpG dinucleotides (CG1–CG4) within the IG-DMR and five CpG dinucleotides (CG5–CG9) within the *MEG3*-DMR. The cytosine residues at the CpG dinucleotides are usually methylated after paternal transmission (filled circles) and unmethylated after maternal transmission (open circles). A 164 bp segment encompassing CG1–CG4 and a 167 bp segment harboring CG5–CG9 were PCR amplified with primer sets (PyF1-PyR1 and PyF2-PyR2) hybridizing to both methylated and unmethylated clones, and sequence primers (SP1 and SP2) were hybridized to single-stranded PCR products. Middle panel: pyrosequencing data in cases 1 and 2, a UPD(14)mat patient, and a control subject. Bottom panel: summary of MIs. (**b**) Microsatellite analysis. The data are consistent with biparental origin of the chromosome 14 pairs. Unequal amplification of the heterozygous peaks in each individual is consistent with short products being more easily amplified than long products, and the patterns of heterozygous peak heights for *D14S292* are comparable between case 1 and the father and between case 2 and the mother, with no disproportionally increased heights of maternally derived peaks.

We first performed pyrosequencing analysis for four CpG dinucleotides (CG1–CG4) within the IG-DMR and five CpG dinucleotides (CG5–CG9) within the *MEG3*-DMR, using bisulfite-treated leukocyte genomic DNA samples (Figure 1). The procedure was as described in the manufacturer's instructions (Qiagen, Valencia, CA, USA). Subsequently, methylation indices (MIs, the ratio of methylated clones) were obtained using PyroMark Q24 (Qiagen). We also studied six UPD(14)mat patients for comparison and 50 control subjects to define the reference ranges of MIs.

When hypomethylation was identified, we performed microsatellite analysis for nine loci on chromosome 14, FISH analysis for the IG-DMR and the *MEG3*-DMR, and array comparative genomic hybridization for the 14q32.2 imprinted region using a custom-build oligo-microarray containing 12 600 probes (Agilent Technologies, Palo Alto, CA, USA).^22^ We also performed pyrosequencing for the *H19*-DMR (ICR1) and the *PEG1*/*MEST*-DMR to re-confirm the absence of the known causes for SRS, and for the KvDMR (ICR2), the *SNRPN*-DMR, the *PLAGL1*-DMR, and the *GNAS* exon A/B-DMR to examine the occurrence of multiple methylation defects.^23^ Primers utilized in this study are shown in Supplementary Table 1.

## Results

### Molecular studies

Pyrosequencing identified hypomethylation of the IG-DMR and the *MEG3*-DMR in two of the 85 SRS patients (cases 1 and 2) (Figure 1). The MIs in case 1 were around the lower limit of the MIs in the six UPD(14)mat patients and much lower than the reference range in the 50 control subjects, whereas the MIs in case 2 were above the maximum MIs in the six UPD(14)mat patients, except for the MI of CG4, and below the reference range in the 50 controls, except for the MI of CG3. The MIs were obviously lower at the *MEG3*-DMR than at the IG-DMR in case 1 and the six UPD(14)mat patients, whereas the MIs were not so different between the IG-DMR and the *MEG3*-DMR in case 2 and the 50 control subjects.

In cases 1 and 2, microsatellite analysis showed biparental transmission of the homologs of chromosome 14, with similar patterns of peak heights for heterozygous alleles between cases and the parents (eg, comparable patterns of peak heights for the 108 bp and the 112 bp alleles of *D14S292* between case 1 and the father and between case 2 and the mother) (Figure 1 and Supplementary Table 2). FISH analysis delineated two copies of the IG-DMR and the *MEG3*-DMR, and array comparative genomic hybridization revealed no discernible copy number alteration in the 14q32.2 imprinted region (Supplementary Figure 1). Furthermore, the MIs for the six DMRs other than the IG-DMR and the *MEG3*-DMR were invariably within the normal range in cases 1 and 2 (Supplementary Table 3).

### Clinical findings of cases 1 and 2

**Figure 2:**
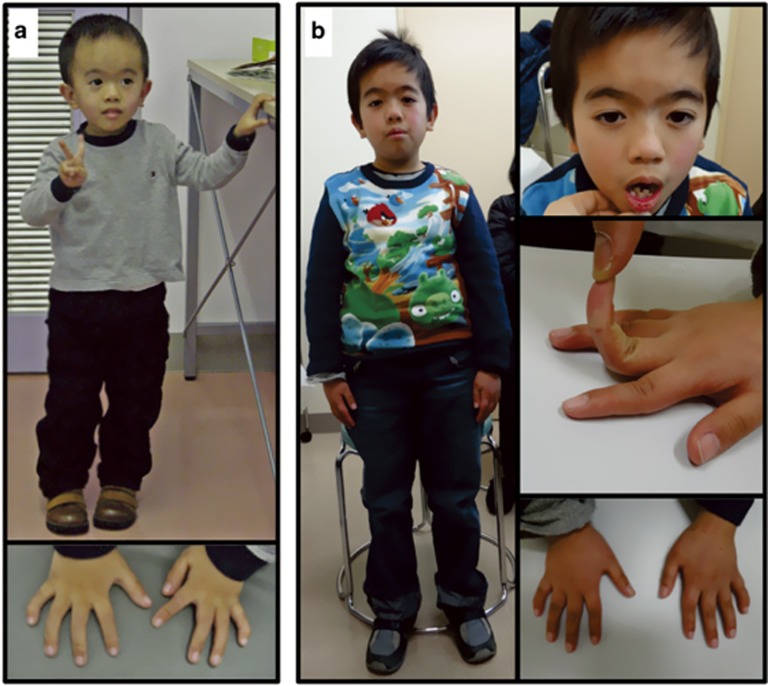
Photographs of case 1. (**a**) At 3 5/12 years of age. He exhibits triangular face with prominent forehead and micrognathia, and clinodactyly of the 5th fingers. (**b**) At 9 6/12 years of age. He exhibits slight central obesity, with the body mass index above the mean. Although this photo suggests mild scoliosis, this is primarily due to body asymmetry with asymmetric leg length. No scoliosis has been identified at the sitting position. He also manifests irregular teeth, joint hypermobility, and clinodactyly of the 5th fingers.

Both cases 1 and 2 showed severe prenatal growth failure, the mandatory criteria (ie, birth length and/or birth weight ≤−2 SD), and four of the five scoring system criteria (ie, relative macrocephaly at birth, postnatal short stature (≤−2 SD) at ≥2 year of age, prominent forehead during early childhood, and body asymmetry) for the diagnosis of SRS, whereas both of them lacked feeding difficulties (Table 1 and Figure 2). In addition, both cases 1 and 2 exhibited triangular face and clinodactyly, and case 1 manifested irregular teeth, brachydactyly, Single palmar crease, muscular hypotonia, and speech delay. Notably, relative macrocephaly with prominent forehead was no longer recognizable with age in both cases. Consistent with this, although the facial appearance was fairly characteristic of SRS in both cases in infancy to early childhood, it became less characteristic in both cases with age (Figure 2).

Both cases 1 and 2 also exhibited TS (UPD(14)mat) clinical features (Supplementary Table 4). In particular, several features characteristic of TS rather than SRS were observed, such as the body mass index above the mean at 9 years of age (though not assessed as obese), joint hypermobility, and small hands in case 1, and small hands and early onset of puberty in case 2.

Clinical survey also revealed that case 2 was born after *in vitro* fertilization-embryo transfer, whereas case 1 was born after natural conception. Furthermore, case 1 was treated with growth hormone for short stature from 6 to 8 years of age, and case 2 received growth hormone therapy for short stature since 5 years of age and gonadotropin-releasing hormone analog therapy for precocious puberty since 7 years of age.

## Discussion

The present study showed that the IG-DMR and the *MEG3*-DMR were severely hypomethylated in case 1 with the MIs comparable to those of UPD(14)mat and moderately hypomethylated in case 2 with the MIs between those of UPD(14)mat patients and those of control subjects, in the absence of UPD(14)mat and microdeletion or copy number alteration involving the DMRs. Furthermore, although such hypomethylation patterns, especially the moderate hypomethylation in case 2, could be caused by post zygotic mosaicism with maternal full or distal 14q segmental isodisomy involving the imprinted region,^24^ microsatellite analysis indicated no disproportionally increased height of the maternally inherited alleles, thereby arguing against the possible mosaicism. Taken together, the results imply the occurrence of epimutation (hypomethylation) of the IG-DMR and the *MEG3*-DMR in cases 1 and 2.

Cases 1 and 2 satisfied SRS diagnostic criteria proposed by Netchine *et al.*^14^ In addition, UPD(14)mat has been identified in a single patient diagnosed as having SRS, although detailed clinical findings are unknown (No. 445 in Table 1).^12^ Furthermore, phenotypic assessment of TS patients with UPD(14)mat reported in the literature reveals that such patients frequently exhibit clinical features utilized as the mandatory and the scoring system criteria for SRS (Table 1). Indeed, pre- and post-natal growth failure, prominent forehead, and feeding difficulties are shared in common by SRS and TS (Table 1 and Supplementary Table 4). In this regard, although the presence or the absence of body asymmetry is not described in most TS patients, it is unlikely that body asymmetry was not reported despite its presence (body asymmetry has been described in a single patient with UPD(14)mat and Prader–Willi Syndrome-like phenotype).^25^ Thus, it is inferred that a considerable degree of phenotypic overlap exists between SRS and TS, except for body asymmetry that is apparently characteristic of SRS, and that epimutations of the IG-DMR and the *MEG3*-DMR were identified in cases 1 and 2 who exceptionally manifested body asymmetry.

Several matters should be pointed out in this study. First, the MIs were obviously lower at the *MEG3*-DMR than at the IG-DMR in case 1 and the six UPD(14)mat patients, whereas the MIs were not so different between the IG-DMR and the *MEG3*-DMR in case 2 and the 50 control subjects. As the IG-DMR and the *MEG3*-DMR function as the imprinting centers in the placenta and the body, respectively,^26^ hypomethylation may be more strictly established in the *MEG3*-DMR of leukocytes in patients with UPD(14)mat and definitive epimutation. Second, multiple methylation defects was not detected in cases 1 and 2. Although the examined DMRs were rather limited, this may argue that isolated epimutation of the IG-DMR and the *MEG3*-DMR can lead to SRS phenotype. Third, relative macrocephaly with prominent forehead became clinically non-recognizable with age in cases 1 and 2. Thus, although clinical features of the two cases were compatible with SRS with no specific finding that serves to distinguish the two cases from other SRS patients in infancy to early childhood, they became less characteristic for SRS with age. Indeed, except for body asymmetry, their recent clinical features were more similar to those of patients with TS^4, 8^ or those of patients with short stature born small-for-date with no catch-up growth.^27^ Such phenotypic change with age, in addition to TS-like clinical features such as recent body mass index gain in case 1 and early onset of puberty in case 2, might be characteristic of SRS patients with an aberrant chromosome 14 imprinted region. Fourth, case 2 was born after *in vitro* fertilization. As *in vitro* fertilization could be a risk factor for the occurrence of epimutation (hypomethylation),^28^ *in vitro* fertilization may be related to the moderate degree of epimutation in case 2. Lastly, epimutation was identified only in two of the 85 SRS patients who were free from epimutation of the *H19*-DMR and UPD(7)mat. Poole *et al*^12^ also have identified UPD(14)mat in one of 127 SRS patients, although clinical assessment remained fragmentary in 127 patients. Thus, UPD(14)mat and related genetic aberrations account for only a small fraction of SRS patients, and underlying factor(s) still remain to be clarified in many SRS patients. Nevertheless, analysis of the chromosome 14 imprinted region is worth attempting in SRS patients, especially in those with neither hypomethylation of the *H19*-DMR nor UPD(7)mat.

In summary, we identified epimutations affecting the IG-DMR and the *MEG3*-DMR in two patients with SRS-compatible phenotype. Further studies will permit to define the phenotypic spectrum of TS with aberrations of the chromosome 14 imprinted region.

## Figures and Tables

**Table 1:** Assessment of Silver–Russell Syndrome (SRS) clinical findings

	*Case 1* *46,XY*	*Case 2* *46,XX*	*No. 445* *...(male)*	*TS patients*	*SRS patients*^a^
*Karyotype genetic cause*	*Epimutation*	*Epimutation*	*UPD(14)mat*	*UPD(14)mat (n=44)*	*Unknown (n=85)*
*SRS diagnosis criteria*^b^					
Mandatory criteria for SRS					
BL and/or BW≤−2 SDS	+	+	+	28/35	85/85
Scoring system criteria for SRS					
Relative macrocephaly at birth^c^	+	+	…	11/21	16/45^d^
PH≤−2 SDS at 2 years	+ (−2.2 SD)	+ (−3.6 SD)	+	21/37	52/61^d^
Prominent forehead	+	+	…	17/21	41/53^d^
Body asymmetry	+	+	−	1/1^e^	19/59^d^
Feeding difficulties	−	−	+	20/25	25/51^d^

*Other findings*					
Gestational age (weeks)	41	37	…	38 (26~42) (*n*=34)	38 (27~41) (*n*=65)
BL cm (SDS)	46.5 (−2.1)	36.5 (−6.0)	…	ND^f^	(−2.9±1.4) (*n*=60)
BW kg (SDS)	2.2 (−2.7)	1.2 (−4.6)	... (−2.6)	ND^f^	(−2.7±1.1) (*n*=64)
BOFC cm (SDS)	32.5 (−0.7)	30.0 (−2.0)	…	ND^f^	(1.9±1.1) (*n*=48)
Present age (years:months)	9:6	9:2	17:9	7:10 (0:3~30:0) (*n*=43)	4:3 (0:1~18:6) (*n*=60)
PH cm (SDS)	120.4 (−2.3)	125.5 (−1.0)^g^	… (0.4 centile)	ND^f^	(−3.2±1.5) (*n*=61)
PW kg (SDS)	26.5 (−0.7)	22.3 (−1.2)	… (0.4 centile)	ND^f^	(−2.8±1.3) (*n*=59)
BMI (kg/m^2^) (SDS)	18.3 (+1.0) SD)	14.2 (−1.1)	…	…	…
POFC cm (SDS)	51.5 (−0.9)	50.3 (−1.5)	…	ND^f^	(−1.8±1.6) (*n*=35)
Relative macrocephaly at present^h^	−	−	…	10/20	29/43
Triangular face	+	+	…	2/12	65/65
Ear anomalies	−	−	…	2/5	15/55
Irregular teeth	+	−	+	2/3	12/45
Clinodactyly	+	+	+	6/6	50/58
Brachydactyly	+	−	−	6/6	34/56
Single palmar crease	+	−	…	7/7	6/49
Muscular hypotonia	+	−	−	29/40	12/50
Speech delay	+	−	−	5/11	18/43

Remark		IVF-ET			
Reference	This study	This study	Poole *et al*^12^	See Supplementary Table S4	Fuke *et al*^13^

Abbreviations: BL, birth length; BMI, body mass index; BOFC, birth occipitofrontal circumference; BW, birth weight; IVF-ET, *in vitro* fertilization-embryo transfer; ND, not determined; PH, present height; POFC, present occipitofrontal circumference; PW, present weight; SDS, standard deviation score; SRS, Silver–Russell Syndrome; TS, Temple Syndrome; UPD(14)mat*,* maternal uniparental disomy 14.

a Japanese SRS patients who have neither epimutation at the *H19*-DMR nor UPD(7)mat.

b The diagnosis of SRS is made when a patient is positive for the mandatory criteria and at least three of the five scoring system criteria (Netchine *et al*^14^)

c BL or BW (SDS)-BOFC (SDS)≤−1.5.

d Of the 85 patients, none have all the five scoring system criteria, 19 exhibit four of the five scoring system criteria, and 66 manifest three of the scoring system criteria.

e The presence of body asymmetry has been documented only in a single patient; while the presence or the absence of body asymmetry is not described, it is inferred that body asymmetry is absent in most, if not all, patients who have been examined for UPD(14)mat.

f Not determined because of lack of precise data in several studies, different growth assessment (SDS or centile) among studies, and different ethnicity.

g The height increase was obviously due to central precocious puberty.

h BL or BW (SDS)-BOFC (SDS)≤−1.5.

For UPD(14)mat and SRS patients, the denominators indicate the number of patients examined for the presence or absence of each feature, and the numerators represent the number of patients assessed to be positive for that feature.In cases 1 and 2 and the 85 SRS patients, birth and present length/height, weight, and occipitofrontal circumference were assessed by the gestational/postnatal age- and sex-matched Japanese reference data from the Ministry of Health, Labor, and Welfare and from the Ministry of Education, Science, Sports and Culture. BMI was evaluated by Japanese reference data.^29^

## References

[bib1] da Rocha ST, Edwards CA, Ito M, Ogata T, Ferguson-Smith AC: Genomic imprinting at the mammalian Dlk1-Dio3 domain. Trends Genet 2008; 24: 306–316. 18471925 10.1016/j.tig.2008.03.011

[bib2] Kagami M, Sekita Y, Nishimura G et al: Deletions and epimutations affecting the human 14q32.2 imprinted region in individuals with paternal and maternal upd(14)-like phenotypes. Nat Genet 2008; 40: 237–242. 18176563 10.1038/ng.2007.56

[bib3] Hoffmann K, Heller R: Uniparental disomies 7 and 14. Best Pract Res Clin Endocrinol Metab 2011; 25: 77–100. 21396576 10.1016/j.beem.2010.09.004

[bib4] Temple IK, Shrubb V, Lever M, Bullman H, Mackay DJ: Isolated imprinting mutation of the DLK1/GTL2 locus associated with a clinical presentation of maternal uniparental disomy of chromosome 14. J Med Genet 2007; 44: 637–640. 17601927 10.1136/jmg.2007.050807 PMC2597958

[bib5] Hosoki K, Ogata T, Kagami M, Tanaka T, Saitoh S: Epimutation (hypomethylation) affecting the chromosome 14q32.2 imprinted region in a girl with upd(14)mat-like phenotype. Eur J Hum Genet 2008; 16: 1019–1023. 18478039 10.1038/ejhg.2008.90

[bib6] Buiting K, Kanber D, Martin-Subero JI et al: Clinical features of maternal uniparental disomy 14 in patients with an epimutation and a deletion of the imprinted DLK1/GTL2 gene cluster. Hum Mutat 2008; 29: 1141–1146. 18454453 10.1002/humu.20771

[bib7] Zechner U, Kohlschmidt N, Rittner G et al: Epimutation at human chromosome 14q32.2 in a boy with a upd(14)mat-like clinical phenotype. Clin Genet 2009; 75: 251–258. 19250383 10.1111/j.1399-0004.2008.01116.x

[bib8] Ioannides Y, Lokulo-Sodipe K, Mackay DJ, Davies JH, Temple IK: Temple Syndrome: improving the recognition of an underdiagnosed chromosome 14 imprinting disorder: an analysis of 51 published cases. J Med Genet 2014; 51: 495–501. 24891339 10.1136/jmedgenet-2014-102396

[bib9] Cassidy SB, Schwartz S, Miller JL, Driscoll DJ: Prader–Willi Syndrome. Genet Med 2012; 14: 10–26. 22237428 10.1038/gim.0b013e31822bead0

[bib10] Mitter D, Buiting K, von Eggeling F et al: Is there a higher incidence of maternal uniparental disomy 14 [upd(14)mat]? Detection of 10 new patients by methylation-specific PCR. Am J Med Genet A 2006; 140: 2039–2049. 16906536 10.1002/ajmg.a.31414

[bib11] Eggermann T: Russell–Silver Syndrome. Am J Med Genet C Semin Med Genet 2010; 154C: 355–364. 20803658 10.1002/ajmg.c.30274

[bib12] Poole RL, Docherty LE, Al Sayegh A et al: Targeted methylation testing of a patient cohort broadens the epigenetic and clinical description of imprinting disorders. Am J Med Genet A 2013; 161: 2174–2182. 10.1002/ajmg.a.36049 23913548

[bib13] Fuke T, Mizuno S, Nagai T et al: Molecular and clinical studies in 138 Japanese patients with Silver–Russell Syndrome. PLoS one 2013; 8: e60105. 23533668 10.1371/journal.pone.0060105 PMC3606247

[bib14] Netchine I, Rossignol S, Dufourg MN et al: 11p15 imprinting center region 1 loss of methylation is a common and specific cause of typical Russell–Silver Syndrome: clinical scoring system and epigenetic-phenotypic correlations. J Clin Endocrinol Metab 2007; 92: 3148–3154. 17504900 10.1210/jc.2007-0354

[bib15] Hitchins MP, Stanier P, Preece MA, Moore GE: Silver–Russell Syndrome: a dissection of the genetic aetiology and candidate chromosomal regions. J Med Genet 2001; 38: 810–819. 11748303 10.1136/jmg.38.12.810 PMC1734774

[bib16] Abu-Amero S, Monk D, Frost J, Preece M, Stanier P, Moore GE: The genetic aetiology of Silver–Russell Syndrome. J Med Genet 2008; 45: 193–199. 18156438 10.1136/jmg.2007.053017

[bib17] Bruce S, Hannula-Jouppi K, Puoskari M et al: Submicroscopic genomic alterations in Silver–Russell Syndrome and Silver–Russell-like patients. J Med Genet 2010; 47: 816–822. 19752157 10.1136/jmg.2009.069427

[bib18] Spengler S, Schonherr N, Binder G et al: Submicroscopic chromosomal imbalances in idiopathic Silver–Russell Syndrome (SRS): the SRS phenotype overlaps with the 12q14 microdeletion Syndrome. J Med Genet 2010; 47: 356–360. 19762329 10.1136/jmg.2009.070052

[bib19] Binder G, Begemann M, Eggermann T, Kannenberg K: Silver–Russell Syndrome. Best Pract Res Clin Endocrinol Metab 2011; 25: 153–160. 21396582 10.1016/j.beem.2010.06.005

[bib20] Gicquel C, Rossignol S, Cabrol S et al: Epimutation of the telomeric imprinting center region on chromosome 11p15 in Silver–Russell Syndrome. Nat Genet 2005; 37: 1003–1007. 16086014 10.1038/ng1629

[bib21] Bliek J, Terhal P, van den Bogaard MJ et al: Hypomethylation of the H19 gene causes not only Silver–Russell Syndrome (SRS) but also isolated asymmetry or an SRS-like phenotype. Am J Hum Genet 2006; 78: 604–614. 16532391 10.1086/502981 PMC1424698

[bib22] Kagami M, Kato F, Matsubara K, Sato T, Nishimura G, Ogata T: Relative frequency of underlying genetic causes for the development of UPD(14)pat-like phenotype. Eur J Hum Genet 2012; 20: 928–932. 22353941 10.1038/ejhg.2012.26 PMC3421115

[bib23] Court F, Martin-Trujillo A, Romanelli V et al: Genome-wide allelic methylation analysis reveals disease-specific susceptibility to multiple methylation defects in imprinting Syndromes. Hum Mutat 2013; 34: 595–602. 23335487 10.1002/humu.22276

[bib24] Yamazawa K, Ogata T, Ferguson-Smith AC: Uniparental disomy and human disease: anoverview. Am J Med Genet C Semin Med Genet 2010; 154C: 329–334. 20803655 10.1002/ajmg.c.30270

[bib25] Berends MJ, Hordijk R, Scheffer H, Oosterwijk JC, Halley DJ, Sorgedrager N: Two cases of maternal uniparental disomy 14 with a phenotype overlapping with the Prader-Willi phenotype. Am J Med Genet A 1999; 84: 76–79. 10.1002/(sici)1096-8628(19990507)84:1<76::aid-ajmg16>3.0.co;2-f 10213052

[bib26] Kagami M, O'Sullivan MJ, Green AJ et al: The IG-DMR and the MEG3-DMR at human chromosome 14q32.2: hierarchical interaction and distinct functional properties as imprinting control centers. PLoS Genet 2010; 6: e1000992. 20585555 10.1371/journal.pgen.1000992 PMC2887472

[bib27] Saenger P, Czernichow P, Hughes I, Reiter EO: Small for gestational age: short stature and beyond. Endocr Rev 2007; 28: 219–251. 17322454 10.1210/er.2006-0039

[bib28] Manipalviratn S, DeCherney A, Segars J: Imprinting disorders and assisted reproductive technology. Fertil Steril 2009; 91: 305–315. 19201275 10.1016/j.fertnstert.2009.01.002 PMC3081604

[bib29] Inokuchi M, Matsuo N, Anzo M, Hasegawa T: Body mass index reference values (mean and SD) for Japanese children. Acta Paediatr 2007; 96: 1674–1676. 17937692 10.1111/j.1651-2227.2007.00490.x

