# Supplementary material for: Epimutations of the IG-DMR and the MEG3-DMR at the 14q32.2 imprinted region in two patients with Silver–Russell Syndrome-compatible phenotype
Source: Eur J Hum Genet. 2014 Nov 5;23(8):1062–7. doi: 10.1038/ejhg.2014.234 (PMC4795120; doi:10.1038/ejhg.2014.234)
Supplement: Supplementary Information [file ejhg2014234x1.pdf]

Supplemental Items
(Supplemental Tables 1-4 and Supplemental Figure 1)

Epimutations of the IG-DMR and the *MEG3*-DMR at the 14q32.2 imprinted region in two patients with Silver-Russell syndrome-compatible phenotype

Masayo Kagami[1*], Seiji Mizuno[2], Keiko Matsubara[1], Kazuhiko Nakabayashi[3], Shinichiro Sano[1], Tomoko Fuke[1], Maki Fukami[1], Tsutomu Ogata[1,4*]

[1]Department of Molecular Endocrinology, National Research Institute for Child Health and Development, Tokyo, Japan

[2]Department of Pediatrics, Central Hospital, Aichi Human Service Center, Aichi, Japan

[3]Department of Maternal-Fetal and Biology, National Research Institute for Child Health and Development, Tokyo, Japan

[4] Department of Pediatrics, Hamamatsu University School of Medicine, Hamamatsu, Japan

Table S1. Primers utilized in the pyrosequencing analysis

	Forward (5' → 3')	Reverse (5' → 3')	Sequence primer	AT	PS
PLAGL -DMR	GGGGTAGTYGTGTTTATAGTTTAG	biotin-CCCAAACACCTACCCTAC	GGGTAGTYGTGTTTATAGTTTAC	55	146
6q24.2	ch6: 144329336-144329359	ch6: 144329214-144329231	ch6: 144329335-144329358		
PEG1 -DMR	GTGTGGTTGGYGGTTTTGGGATTA	biotin-ACACCCCCTCCTCAAATA	TGTTTTTGGGYGAAAATTTTAT	55	143
7q32.2	ch7: 130132206-130132229	ch7: 130132332-130132348	ch7: 130132276-130132297		
H19 -DMR	GAGTTYGGGGGTTTTTGTATAGT	biotin-TAAATAATACCCRACCTAAAAATCTAA	GGTTGTAGTTGTGGAAT	54	244
11p15.5	ch11: 2021685-2021713	ch11: 2021901-2021928	ch11: 2021748-2021765		
KvDMR	GGATTTAGAATTAYGATGYGGATTTTA	biotin-TCCCATCTACACCTTATAAACA	TTTTGAATTATTATGAGAATTAT	55	155
11p15.5	ch11: 2720333-2720359	ch11: 2720466-2720487	ch11: 2720383-2720407		
IG-DMR	ATTTGGTATTTGTAGTTTTATGTTAAGAT	biotin-AATCAAAACAACTCAAATCCTTTATAAC	AATTGGGTTTGTTAGTAG	54	164
14q32.2	ch14: 101275613-101275642	ch14: 101275749-101275776	ch14: 101275685-101275702		
MEG3 -DMR	TTGTGTTTGAATTTATTTTGTTT	biotin-CCCCAAATTCTATAACAAATTACTCT	GTGTTTGAATTTATTTTGTTT	54	167
14q32.2	ch14: 101292170-101292192	ch14: 101292311-101292336	ch14: 101292172-101292192		
SNRPN -DMR	TGGGGTTTTAGGGGTTTAG	biotin-AATAAAAATAACCCCTCCCCAAACTATCT	GAGTTTGGAGTAGAGTGGA	60	306
15q11.2	ch15: 25199961-25199979	ch15: 25200236-25200266	ch15: 25200125-25200143		
GNAS exon A/B-DMR	GGTTTTTYGTTGTTGTTGGGTGTT	biotin-CCTAACCCRAATCCCTACTTAC	AATTTTTAGGTAGTTAGTTTAGT	54	229
20q13.32	ch20: 57464673-57464696	ch20: 57464879-57464901	ch20: 57464746-57464769		

AT: annealing temperature (°C); PS: product size (bp); Y: C or T (pyrimidine); and R: A or G (purine).

Physical positions of the primers are based on the NCBI database (Genome Build 37.1).

Table S2. The results of microsatellite analysis.

Locus	Position	Case 1	Mother	Father	Assessment	Case 2	Mother	Father	Assessment
D14S80	14q12	98/106	98/106	98/104	Biparental*	96/98	98/106	96/104	Biparental
D14S608	14q12	205/213	209/213	197/205	Biparental	197/213	197/209	213/225	Biparental
D14S588	14q23-24.1	114/126	114/118	122/126	Biparental	114/130	114/130	114	Biparental*
D14S1000	14q31.1	136/138	126/138	136/140	Biparental	126/136	126	126/136	Biparental†
D14S617	14q32.12	139/161	161/165	139/153	Biparental	139/143	143/165	139/161	Biparental
D14S1006	14q32.2	136/138	126/138	136/140	Biparental	126/136	126	126/136	Biparental†
D14S985	14q32.2	137	137	129/137	N.I.	129/145	129/145	129	Biparental*
D14S1010	14q32.33	146/148	144/146	144/148	Biparental	144/150	136/144	140/150	Biparental
D14S292	14q32.33	108/112	112	108/112	Biparental†	108/112	108/112	112/114	Biparental*

* The possibility of segmental maternal heterodisomy is not postulated, because co-existence of maternal heterodisomic loci and biparental loci can not occur.

† Since paternal heterodisomy is not assumed, the results indicate biparental transmission.

N.I.: not informative.

D14S985 resides on intron 3 of *MEG3*.

Table S3. Methylation indices (%) for CpG dinucleotides at disease-associated DMRs

	CpG	Case 1	Case 2	Controls (n=50)
				Median (Minimum ~ Maximum)
H19 -DMR	10	51	41	48 (37 ~ 60)
(Ch. 11p15.5)	11	51	45	50 (39 ~ 64)
	12	49	40	46 (36 ~ 57)
	13	49	40	45 (36 ~ 55)
PEG1 /*MEST*- DMR	14	69	62	60 (56 ~ 70)
(Ch. 7q32.2)	15	68	63	59 (55 ~ 69)
	16	66	59	58 (52 ~ 68)
	17	70	64	61 (42 ~ 73)
	18	65	59	57 (47 ~ 66)
	19	72	62	60 (54 ~ 70)
KvDMR	20	49	54	58 (49 ~ 66)
(Ch. 11p15.5)	21	52	55	61 (52 ~ 68)
	22	44	49	48 (41 ~ 54)
	23	46	52	48 (42 ~ 55)
	24	55	56	67 (55 ~ 72)
	25	52	58	64 (55 ~ 71)
SNRPN -DMR	26	39	41	42 (36 ~ 47)
(Ch. 15q11.2)	27	38	41	43 (36 ~ 48)
	28	40	42	44 (36 ~ 50)
	29	39	42	44 (37 ~ 48)
	30	36	38	38 (32 ~ 42)
	31	40	42	42 (36 ~ 47)
PLAGL1 -DMR	32	51	48	47 (31 ~ 52)
(Ch. 6q24.2)	33	47	45	45 (27 ~ 51)
	34	45	44	48 (40 ~ 56)
	35	37	37	39 (31 ~ 47)
	36	47	45	50 (40 ~ 58)
	37	45	43	49 (37 ~ 55)
	38	50	48	53 (41 ~ 58)
GNAS exon A/B-DMR	39	45	42	42 (37 ~ 46)
(Ch. 20q13.32)	40	42	42	41 (38 ~ 47)
	41	43	42	41 (37 ~ 46)
	42	38	37	36 (31 ~ 41)
	43	42	43	41 (36 ~ 46)
	44	35	34	34 (30 ~ 38)
	45	42	41	41 (38 ~ 49)
	46	35	34	33 (30 ~ 37)
	47	39	37	36 (32 ~ 42)
	48	40	39	37 (35 ~ 44)
	49	47	45	45 (42 ~ 50)

CpG1–4 reside in the IG-DMR, and CpG5–9 are located in the *MEG3* -DMR (see Figure 1).

Table S4. Assessment of UPD(14)mat clinical features

	Case 1	Case 2	No. 445	Temple syndrome
Karyotype	46,XY	46,XX	… (Male)	
Genetic cause	Epimutation	Epimutation	UPD(14)mat	UPD(14)mat (n=44)
Present age (years:months)	9:6	9:2	17:9	7:10 (0:3~30:0) (n=43)
Sex	Male	Female	Male	M:F=21:23
Karyotype	46,XY	46,XX	…	Normal:Abnormal=15:29
Premature delivery	–	–	–	13/36
Gestational age (wks)	41	37	…	38 (26~42) (n=34)
Prenatal growth failure	+	+	+	28/35
Postnatal growth failure	+	+	+	21/37
Early onset of puberty	–	+	…	13/22
Menarche (years:months)	…	+ (8:8)[a]	…	8:11 (8~11) (n=5)
Feeding difficulties	–	–	+	20/25
Mental retardation	–	–	–	14/37
Obesity	–	–	–	9/32
BMI (kg/m^2) (SDS)	18.3 (+1.0)	14.2 (–1.1)	…	…
Muscular hypotonia	+	–	–	29/40
Scoliosis	–	–	…	7/29
Joint hypermobility	+	...	…	14/30
Small hands	+	+	…	33/38
Prominent forehead	+	+	…	17/21
Recurrence otitis media	–	+	…	9/22
Reference	This study	This study	1	2–30 and nine our unpublished patients

BMI: body mass index; and SDS standard deviation score.

[a] Menarchial age in normal Japanese girls: 12.25 ± 1.25 years. Case 2 has been treated with gonadotropin-releasing hormone analog since 7 years and eight months of age, because of central precocious puberty with breast development.

References

1. Poole RL, Docherty LE, Al Sayegh A *et al*: Targeted methylation testing of a patient cohort broadens the epigenetic and clinical description of imprinting disorders. *Am J Med Genet A* 2013; **161**: 2174-2182.
2. Temple IK, Cockwell A, Hassold T *et al*: Maternal uniparental disomy for chromosome 14. *J Med Genet* 1991; **28**: 511-14.
3. Pentao L, Lewis RA, Ledbetter DH *et al*: Maternal uniparental isodisomy of chromosome 14: association with autosomal recessive rod monochromacy. *Am J Hum Genet* 1992; **50**: 690-699.
4. Antonarakis SE, Blouin JL, Maher J *et al*: Maternal uniparental disomy for human chromosome 14, due to loss of a chromosome 14 from somatic cells with t(13;14) trisomy 14. *Am J Hum Genet* 1993; **52**: 1145-1152.
5. Barton DE, McQuaid S, Stallings R *et al*: Further evidence for an emerging maternal uniparental disomy chromosome 14 syndrome: analysis of a phenotypically abnormal de novo Robertsonian translocation t(13;14) carrier. *Am J Hum Genet* 1993; **59** (Suppl): 687.
6. Healey S, Powell F, Battersby M *et al*: Distinct phenotype in maternal uniparental disomy of chromosome 14. *Am J Med Genet* 1994; **51**: 147-149.
7. Coviello DA, Panucci E, Mantero MM *et al*: Maternal uniparental disomy for chromosome 14. *Acta Genet Med Gemellol (Roma)* 1996; **45**: 169-172.
8. Tomkins DJ, Roux AF, Waye J *et al*: Maternal uniparental isodisomy of human chromosome 14 associated with a paternal t(13q14q) and precocious puberty. *Eur J Hum Genet* 1996; **4**: 153–159.
9. Link L, McMilin K, Popovich B *et al*: Maternal uniparental disomy for chromosome 14. *Am J Hum*

Genet 1996; **59** (Suppl): 687.
10. DesiletsVA, Young SL, Kalousek DK *et al*: Maternal uniparental disomy for chromosome 14. *Am J Hum Genet* 1997; **61** (Suppl): 691.
11. Robinson WP, Barrett IJ, Bernard L *et al*: Meiotic origin of trisomy in confined placental mosaicism is correlated with presence of fetal uniparental disomy, high levels of trisomy in trophoblast, and increased risk of fetal intrauterine growth restriction. *Am J Hum Genet* 1997; **60**: 917-927.
12. Splitt MP, Goodship JA. Another case of maternal uniparental disomy chromosome 14 syndrome. *Am J Med Genet* 1997; **72**: 239-240.
13. Harrison KJ, Allingham-Hawkins DJ, Hummel J *et al*: Risk of uniparental disomy in Robertsonian translocation carriers: identification of upd(14) in a small cohort. *Am J Hum Genet* 1998; **63** (Suppl): 51.
14. Miyoshi O, Hayashi S, Fujimoto M *et al*: Maternal uniparental disomy for chromosome 14 in a boy with intrauterine growth retardation. *J Hum Genet* 1998; **43**: 138-142.
15. Berends MJ, Hordijk R, Scheffer H *et al*: Two cases of maternal uniparental disomy 14 with a phenotype overlapping with the Prader-Willi phenotype. *Am J Med Genet* 1999; **84**: 76-79.
16. Fokstuen S, Ginsburg C, Zachmann M *et al*: Maternal uniparental disomy 14 as a cause of intrauterine growth retardation and early onset of puberty. *J Pediatr* 1999; **134**: 689-695.
17. Hordijk R, Wierenga H, Scheffer H *et al*: Maternal uniparental disomy for chromosome 14 in a boy with a normal karyotype. *J Med Genet* 1999; **36**: 782-785.
18. Manzoni MF, Pramparo T, Stroppolo A *et al*: A patient with maternal chromosome 14 UPD presenting with a mild phenotype and MODY. *Clin Genet* 2000; **57**: 406-408.
19. Sanlaville D, Aubry MC, Dumez Y *et al*: Maternal uniparental heterodisomy of chromosome 14: chromosomal mechanism and clinical follow up. *J Med Genet* 2000; **37**: 525-528.
20. Eggermann T, Mergenthaler S, Eggermann K *et al*: Identification of interstitial maternal uniparental disomy (UPD) (14) and complete maternal UPD(20) in a cohort of growth retarded patients. *J Med Genet* 2001; **38**: 86-89.
21. Papenhausen P, Wylie A, Shah H *et al*: Clinical/molecular studies and a diagnostic reversal. *Am J Hum Genet* 2001; **69** (Suppl): 760.
22. Towner DR, Shaffer LG, Yang SP *et al*: Confined placental mosaicism for trisomy 14 and maternal uniparental disomy in association with elevated second trimester maternal serum human chorionic gonadotrophin and third trimester fetal growth restriction. *Prenat Diagn* 2001; **21**: 395-398.
23. Worley KA, Rundus VR, Lee EB *et al*: Maternal uniparental disomy 14 presenting as language delay. *Am J Hum Genet* 2001; **69** (Suppl): 738.
24. Giunti L, Lapi S, Guarducci S, *et al*: Maternal heterodisomy for chromosome 14 and 13/14 Robertsonian translocation in a female with normal development, short stature, and dysmorphic features. *Eur J Hum Genet* 2002; **10** (Suppl): 214.
25. Kayashima T, Katahira M, Harada N *et al*: Maternal isodisomy for 14q21-q24 in a man with diabetes mellitus. *Am J Med Genet* 2002; **111**: 38-42.
26. Cox H, Bullman H, Temple IK. Maternal UPD(14) in the patient with a normal karyotype: clinical report and a systematic search for cases in samples sent for testing for Prader-Willi syndrome. *Am J Med Genet A* 2004; **127A**: 21-25.
27. Aretz S, Raff R, Woelfle J *et al*: Maternal uniparental disomy 14 in a 15-year-old boy with normal karyotype and no evidence of precocious puberty. *Am J Med Genet A* 2005; **135**: 336-338.
28. Falk MJ, Curtis CA, Bass NE *et al*: Maternal uniparental disomy chromosome 14: case report and literature review. *Pediatr Neurol* 2005; **32**: 116-120.
29. Takahashi I, Takahashi T, Utsunomiya M *et al*: Long-acting gonadotropin-releasing hormone analogue treatment for central precocious puberty in maternal uniparental disomy chromosome 14. *Tohoku J Exp Med* 2005; **207**: 333-338.
30. Mitter D, Buiting K, von Eggeling F *et al*: Is there a higher incidence of maternal uniparental disomy 14 [upd(14)mat]? Detection of 10 new patients by methylation-specific PCR. *Am J Med Genet A* 2006; **140**: 2039-2049.

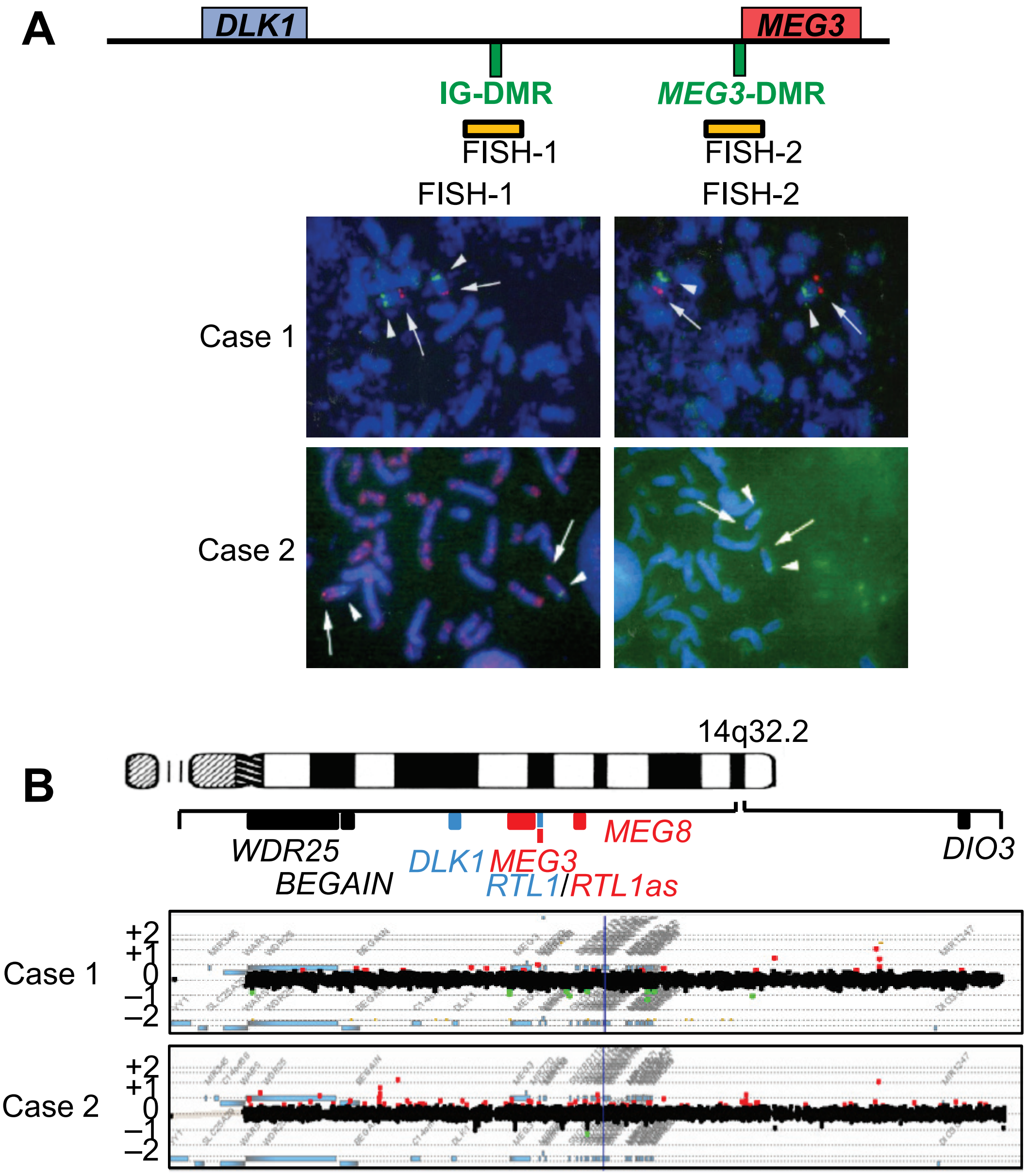

Figure S1. FISH and array CGH analyses in cases 1 and 2.

A. FISH analysis for the IG-DMR and the *MEG3*-DMR. A long PCR product of 5,104 bp encompassing the IG-DMR is utilized as FISH probe 1, and a long PCR product of 5,182 bp encompassing the *MEG3*-DMR is utilized as FISH probe 2. Two red signals are identified by FISH probe 1 and FISH probe 2 in cases 1 and 2, together with two green signals derived from an RP11-566I2 probe for 14q12 used as an internal control.

B. Array CGH analysis for the 14q32.2 imprinted region. The black, the red, and the green dots denote signals indicative of the normal, the increased (> +0.5), and the decreased (< –1.0) copy numbers, respectively. Although several red and green signals are seen, there is no portion associated with ≥3 consecutive red or green signals.
